# Unilateral brucella epidydimo-orchitis: a case report and literature review

**DOI:** 10.1093/jscr/rjag378

**Published:** 2026-05-27

**Authors:** Mohannad N AbuHaweeleh, Mohamed Abdelkareem, Nabil Mahmood, Tawiz Gul, Béla Tállai, Morshed Salah

**Affiliations:** College of Medicine, QU Health, Qatar University, Doha, Qatar; Department of Urology, Ambulatory Care Center, Hamad Medical Corporation, Doha, Qatar; Urology Section, Surgery Department, Hazm Mebaireek General Hospital, Hamad Medical Corporation, Doha, Qatar; Department of Radiology, Hazm Mebaireek General Hospital, Hamad Medical Corporation, Doha, Qatar; Urology Section, Surgery Department, Hazm Mebaireek General Hospital, Hamad Medical Corporation, Doha, Qatar; Urology Section, Surgery Department, Hazm Mebaireek General Hospital, Hamad Medical Corporation, Doha, Qatar; Urology Section, Surgery Department, Hazm Mebaireek General Hospital, Hamad Medical Corporation, Doha, Qatar

**Keywords:** epididymo-orchitis, brucellosis, brucella epididymo-orchitis, genitourinary infections

## Abstract

Brucellosis is a zoonotic infection transmitted through contact with infected animals or consumption of unpasteurized dairy products and remains endemic in regions such as the Mediterranean, Middle East, Asia, and Africa. Its variable clinical manifestations often mimic other diseases, complicating diagnosis. We report a case of unilateral Brucella epididymo-orchitis in a middle-aged animal handler to highlight diagnostic and therapeutic challenges. A 32-year-old Indian man working with camels and goats presented with fever, body aches, fatigue, poor appetite, and a lip ulcer. Initial examination was unremarkable. Laboratory tests showed hypochromic microcytic anemia, leukocytosis, and positive Brucella serology. Scrotal ultrasonography demonstrated testicular enlargement with increased vascularity, while blood cultures grew *Brucella canis*. Although treatment adherence was initially poor due to medication side effects, clinical and laboratory parameters improved after completing antibiotics. The patient was discharged on doxycycline and cefuroxime with close follow-up.

## Introduction

Brucellosis is a multisystem disease with a broad clinical spectrum that often mimics other conditions, creating significant diagnostic challenges [1]. Its nonspecific presentation frequently leads to misdiagnosis or delayed diagnosis, particularly in endemic regions where human–animal contact is common. These challenges are exacerbated in resource-limited settings with restricted access to accurate diagnostic tools, resulting in underestimation of disease burden and inadequate public health responses. Conversely, in settings relying on less specific tests, overdiagnosis may occur, increasing public concern unnecessarily [2]. Brucella orchitis, a recognized but underreported complication, remains insufficiently described in the literature, with limited case studies available. Its clinical overlap with other genitourinary conditions further complicates diagnosis and delays appropriate management. Additionally, there is no clear consensus on optimal treatment strategies, and current approaches lack robust clinical validation. Given these gaps, further research is needed to better characterize its presentation and management. Here, we present a case of unilateral Brucella epididymo-orchitis in a middle-aged man.

## Case presentation

A 32-year-old male from India presented to the clinic following multiple visits over a short period. His initial complaint included a 3-day history of fever, body aches, fatigue, and diminished appetite. Additionally, he noticed the development of an ulcer on his lip 1 week prior. He denied any other symptoms. His past medical, surgical, and family histories were unremarkable. The patient is employed as an animal caregiver, working with camels and goats. On physical examination, no significant findings were noted except for a fever of 38°C. He was prescribed 1 g of paracetamol for the fever, along with topical triamcinolone and vitamin B complex for the aphthous ulcer.

## Methods

### Investigations

**Figure 1:**
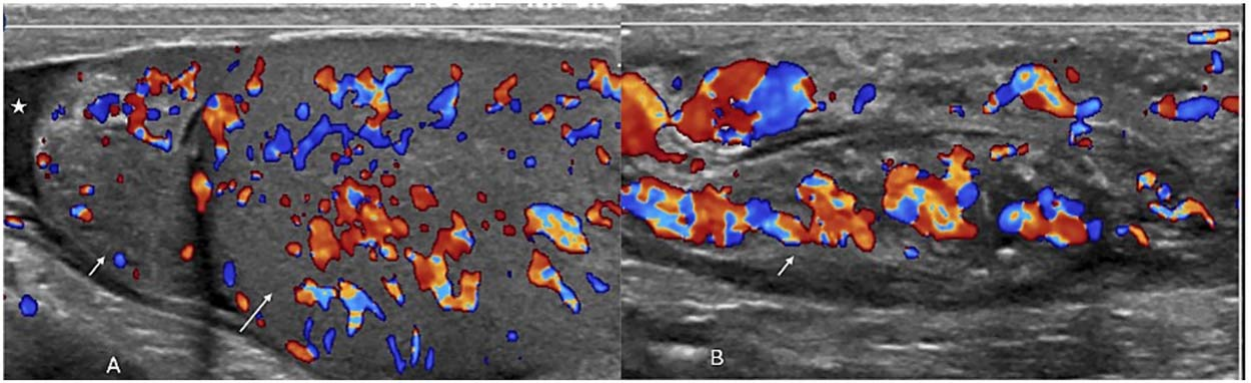
Initial color doppler examination of the scrotum reveals mildly enlarged epididymis (short arrow in A) and testis (long arrow in A) with increased vascularity along with mild hydrocele (star in A). At a superior level, the spermatic cord was found to be thickened showing increased vascularity as well (short arrow in B).

The patient returned 3 days after his initial visit with persistent fever, and laboratory findings (Table 1) supported a diagnosis of acute brucellosis, for which doxycycline 100 mg daily for 6 weeks was initiated. Eight days later, he presented with abdominal pain, nausea, vomiting, and subjective fever, and was treated symptomatically with domperidone, scopolamine, and paracetamol. Eight weeks after the initial visit, he presented to the emergency department with left hemiscrotal pain and swelling following minor trauma, along with intermittent fever and body aches; he admitted poor antibiotic compliance and prior ingestion of unpasteurized milk. Examination showed mild left scrotal swelling and tenderness with fever (39.1°C), and blood cultures grew Gram-negative coccobacilli suggestive of *Brucella canis*, prompting notification of infection control. Doppler ultrasound demonstrated a normal right testis, while the left testis had increased vascularity with a bulky epididymis and spermatic cord and minimal hydrocele (Fig. 1). Chest X-ray and echocardiography were unremarkable, and the patient was admitted for conservative management.

**Table 1:** Laboratory investigations at the initial visit and 8 weeks later

Test	Initial visit	8 weeks later	Reference range
White blood cell count (WBC)	7.4 x 10^3^/μl	11.5 x 10^3^/μl	4–10 x 10^3^/μl
Hemoglobin (Hgb)	12.8 g/dl	11.5 g/dl	13–17 g/dl
Hematocrit (Hct)	37.1%	33.4%	40–50%
Mean corpuscular volume (MCV)	76.8 fl	74.2 fl	83–101 fl
Mean corpuscular hemoglobin (MCH)	26.5 pg	25.6 pg	27–32 pg
Mean corpuscular hemoglobin concentration (MCHC)	34.6 g/dl	34.4 g/dl	32.5–34.5 g/dl
Platelet distribution width (PDW)		11.8 fl	9.4–10.6 fl
Absolute neutrophil count		7.8 x 10^3^/μl	2–7 x 10^3^/μl
Monocyte count		1.1 x 10^3^/μl	0.2–1 x 10^3^/μl
Mean platelet volume (MPV)	7.1 fl		7.4–10.4 fl
Sodium (Na ^+^)	133 mmol/L	130 mmol/L	135–145 mmol/L
Chloride (Cl ^−^)	94.9 mmol/L		96–110 mmol/L
C-reactive protein (CRP)	27.4 mg/L	94.7 mg/L	0–5 mg/L
ALT (alanine aminotransferase)		44 U/L	0–41 U/L
AST (aspartate aminotransferase)		42 U/L	0–40 U/L
Blood culture result		Positive for *Brucella canis*	−
Brucella Ab IgG	Positive		−
Brucella Ab IgM	Positive		−
*Brucella abortus* titer	1:1280		−
*Brucella melitensis* titer	1:1280		−
Urine pH	6.5		−
Urine specific gravity	1.01		−
Urine bilirubin	Positive		−
Urine urobilinogen	Positive		−
Urine leukocytes	++		−
Urine nitrites	Negative		−
Urine protein	Negative		−
Urine ketones	Negative		−
Urine erythrocytes	Negative		−
Urine glucose	Normal		−

**Figure 2:**
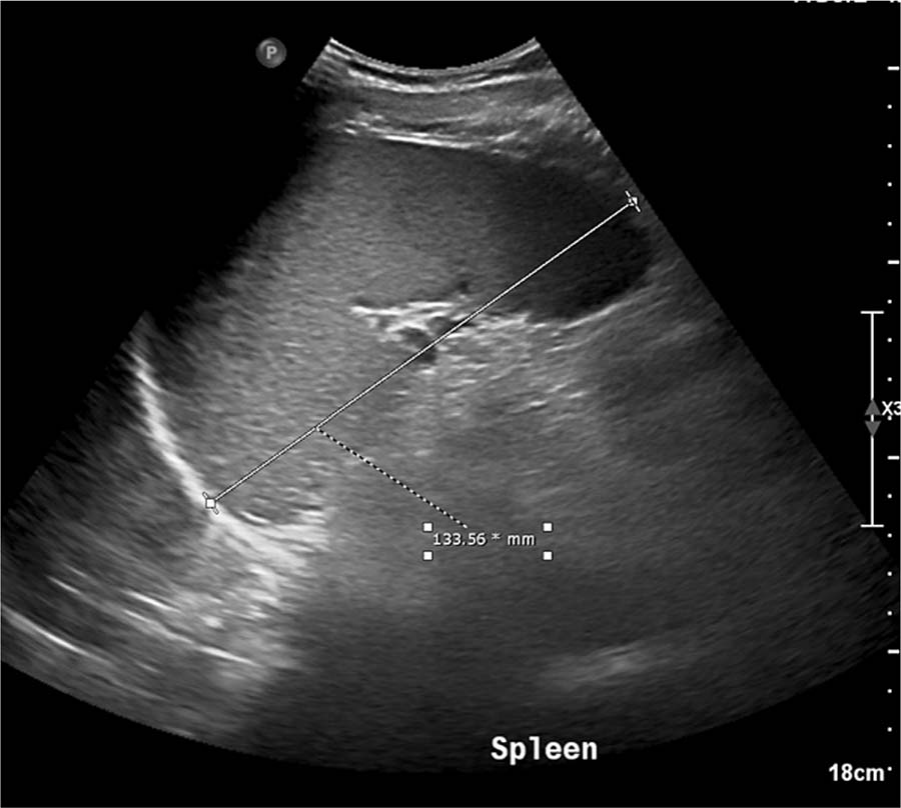
Grey scale ultrasound examination of the abdomen showed a mildly enlarged spleen.

The following day, he remained febrile (39.5°C) with persistent pain; infectious disease team recommended adding gentamicin. Abdominal ultrasound revealed splenomegaly (13.4 cm) and mildly increased liver echogenicity (Fig. 2). Laboratory findings were largely consistent with prior results, with additional abnormalities including low creatinine, low bicarbonate, hypoalbuminemia, and mildly elevated liver enzymes. Iron studies indicated iron deficiency anemia, and supplementation was started, supported by findings of low serum iron, total iron-binding capacity (TIBC), transferrin, and iron saturation with elevated ferritin. Peripheral smear showed mild hypochromic microcytic anemia with occasional abnormal red cell morphology, mild neutrophilic leukocytosis with toxic changes, and normal platelet count.

### Intervention

**Figure 3:**
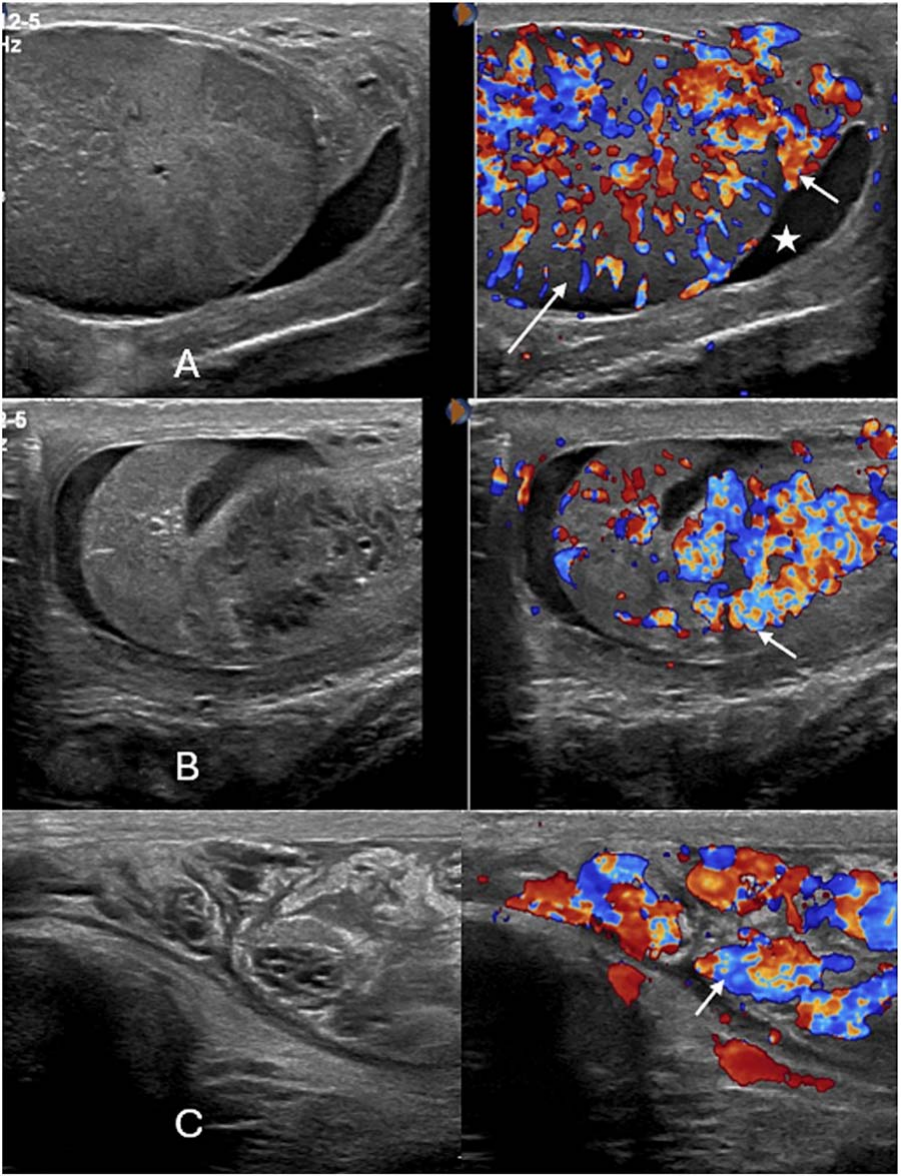
Follow up color doppler examination of the scrotum reveals progressively enlarged epididymis (short arrow in A and B) and testis (long arrow in A) with increased vascularity along with mild hydrocele (star in A). At a superior level, the spermatic cord also shows progressive thickening and increased vascularity (short arrow in C).

The patient presented with left hemiscrotal pain, fever (38°C), and gastrointestinal symptoms; examination revealed a bulky, tender left epididymis with hydrocele, and findings were consistent with brucella epididymo-orchitis based on ultrasonography. The urology team recommended adding piperacillin/tazobactam (Tazocin) 4.5 g every 8 hours and repeat imaging if no improvement. Laboratory results showed normalization of WBC (8.4 × 10^3^/μl) but persistently elevated c-reactive protein (CRP) (171.4 mg/L), with aspartate aminotransferase (AST) normalized and alanine aminotransferase (ALT) still elevated. The following day, the patient was afebrile with intermittent pain, and Tazocin was discontinued per infectious disease advice. Repeat Doppler ultrasound demonstrated a normal right testis and an enlarged, hypervascular left testis with heterogeneous epididymis and mild hydrocele containing floating echogenic foci (Fig. 3). The patient subsequently showed progressive clinical and biochemical improvement, with decreasing CRP (151.5 → 41.5 mg/L), reduced inflammation, and resolution of pain. He was discharged in stable condition on doxycycline 100 mg daily for 6 weeks, cefuroxime 500 mg BID for 5 days, and ferrous fumarate 200 mg daily for 30 days, with follow-up appointments arranged. At 1-week review, he remained asymptomatic with significant recovery and no new complaints.

## Discussion

We report a case of a middle aged animal handler who presented with non-specific symptoms complicated later with unilateral brucella epidiymo-orchitis.

Brucellosis is a multisystem disease with variable presentations ranging from mild to severe, classified as acute (0–2 months), subacute (2–12 months), or chronic (>12 months) [2, 3]. It commonly presents with nonspecific symptoms such as fever, sweating, chills, nausea, vomiting, myalgia, and osteoarticular involvement, while genitourinary manifestations like scrotal pain and swelling may indicate Brucella orchitis [4]. In our case, a lip ulcer was noted but not explored diagnostically, though such findings may reflect systemic involvement. Brucellosis in males can cause multiple genitourinary complications, including epididymo-orchitis, prostatitis, cystitis, and renal involvement, and may mimic testicular tumors or recur, complicating management [5]. Genitourinary symptoms often include unilateral scrotal swelling, pain, dysuria, and urethral discharge, though urinary abnormalities are typically mild and present in only a minority of patients [3, 6].

Delayed diagnosis of orchitis worsens outcomes, increasing the risk of complications such as abscess formation, infarction, necrosis, and even spontaneous rupture [7, 8]. Norton et al. reported complications in 39% of acute epididymo-orchitis cases, highlighting the importance of early treatment [7]. In Brucella epididymo-orchitis, long-term complications are generally uncommon but clinically relevant, with infertility rates ranging from ~0.3% in large series to 23.5% in smaller studies, likely due to differences in study design and disease factors [9, 10]. Chronic scrotal pain and testicular atrophy may occur in a minority of cases, particularly with severe or delayed disease [11], though overall prognosis is favorable with timely and appropriate therapy.

## Conclusion

Brucella orchitis poses diagnostic challenges due to clinical overlap with testicular infections and malignancy. Our case demonstrated atypical features requiring differential diagnosis. A high index of suspicion is essential, in endemic regions or at-risk populations. Early incorporation of Brucella serology in treatment-resistant epididymo-orchitis improves diagnostic accuracy and facilitates timely management.

## Consent

Written informed consent was obtained from the patient for publication of this case report and any accompanying images.

## Learning points

- Brucella epididymo-orchitis is a rare but important genitourinary complication of brucellosis and should be considered in patients from endemic regions presenting with scrotal pain, fever, and systemic symptoms.
- Occupational exposure to livestock and consumption of unpasteurized dairy products are key epidemiological risk factors that should prompt early consideration of brucellosis in the differential diagnosis.
- Brucella epididymo-orchitis may clinically mimic other causes of epididymo-orchitis or testicular malignancy, making a high index of clinical suspicion essential for accurate diagnosis.
- Serological testing and blood cultures are important diagnostic tools, while scrotal ultrasonography with Doppler helps identify inflammatory changes and exclude other testicular pathologies.
- Early diagnosis and appropriate antibiotic therapy are crucial to prevent complications such as abscess formation, testicular infarction, and long-term fertility issues.

## Data Availability

Data sharing is not applicable – no new data generated.
